# Incorrect interpretation of carbon mass balance biases global vegetation fire emission estimates

**DOI:** 10.1038/ncomms11536

**Published:** 2016-05-05

**Authors:** N. C. Surawski, A. L. Sullivan, S. H. Roxburgh, C.P. Mick Meyer, P. J. Polglase

**Affiliations:** 1 CSIRO Agriculture, GPO Box 1700, Canberra, Acton 2601, Australian Capital Territory, Australia; 2 CSIRO Land and Water, GPO Box 1700, Canberra, Acton 2601, Australian Capital Territory, Australia; 3 CSIRO Oceans and Atmosphere, 107–121 Station Street, Aspendale, Victoria 3195, Australia

## Abstract

Vegetation fires are a complex phenomenon in the Earth system with many global impacts, including influences on global climate. Estimating carbon emissions from vegetation fires relies on a carbon mass balance technique that has evolved with two different interpretations. Databases of global vegetation fire emissions use an approach based on ‘consumed biomass', which is an approximation to the biogeochemically correct ‘burnt carbon' approach. Here we show that applying the ‘consumed biomass' approach to global emissions from vegetation fires leads to annual overestimates of carbon emitted to the atmosphere by 4.0% or 100 Tg compared with the ‘burnt carbon' approach. The required correction is significant and represents ∼9% of the net global forest carbon sink estimated annually. Vegetation fire emission studies should use the ‘burnt carbon' approach to quantify and understand the role of this burnt carbon, which is not emitted to the atmosphere, as a sink enriched in carbon.

## Abstract (web summary)

Vegetation fires contribute to global carbon emissions, but uncertainty exists due to inconsistencies in the treatment of post-burn combustion. Here, it is shown that the ‘consumed biomass' approach overestimates emissions by 4%, which can be corrected using an alternative ‘burnt carbon' method.

## Graphical abstract

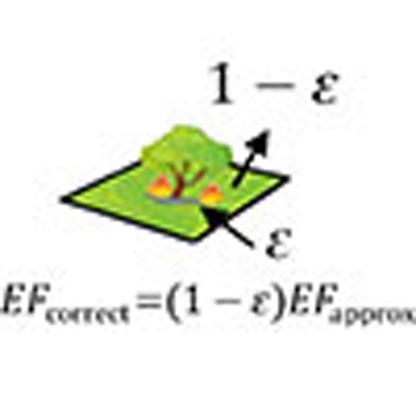

Vegetation fires have existed in the Earth system since shortly after the appearance of terrestrial vegetation ∼420 million years ago^1^,^2^ and are a necessary disturbance for maintaining some ecosystems^3^. They also have a range of anthropogenically deleterious consequences such as damage to assets and infrastructure, loss of life, as well as degrading ambient air quality leading to negative impacts on human health^4^. Vegetation fires also perturb global biogeochemical cycles, with impacts on climate^4^,^5^,^6^. It is estimated that over the period 2001–2010, global vegetation fires emitted an average of 2.5 Pg per year (1 Pg=1 × 10^15^ g) of carbon to the atmosphere^7^, equivalent to 30% of annual-averaged anthropogenic emissions from fossil fuel combustion over 2004–2013 (ref. 8). Changes in global vegetation fire regimes, such as an increased fire frequency, have been predicted from modelling studies undertaken in the northwest of the United States^9^, as well as Mediterranean-type ecosystems under a warmer and wetter future climate^10^. Furthermore, charcoal records from boreal forests suggest that current vegetation fire frequencies are at their highest level in the last 3,000 years^11^ and predictions of a future increase in fire intensity have been made^12^. Given the global impact of vegetation fires on ecosystems and climate, accurate accounting methods are required to estimate their greenhouse gas emissions, to characterize their climate forcing and to properly design carbon mitigation strategies involving the management of vegetation.

Using the Intergovernmental Panel on Climate Change (IPCC) methodology^13^, total emissions from fire are calculated using the product of activity and an emissions factor (EF). Activity refers to a process occurring in the environment that leads to emissions (for example, fire) and an EF is thus defined as emissions per unit activity.

When calculating an EF from fire, the principle of a carbon mass balance (CMB) is invoked, with the technique ultimately being attributed to either Ward *et al*.^14^ or Radke *et al*.^15^. While the partitioning of the chemical species of interest and total emitted carbon into reference-gas-normalized emission ratios is consistently applied in vegetation fire emission studies^16^,^17^,^18^, there have been inconsistencies in the treatment of vegetation exposed to fire but not emitted to the atmosphere (that is, post-burn combustion residue).

In this article, we show that neglecting changes in the post-burn carbon content (CC_post_, %) leads to an upward bias in emission estimates for the ‘consumed biomass' CMB interpretation compared with the ‘burnt carbon' approach. We therefore recommend that vegetation fire studies employ the ‘burnt carbon' approach to more accurately estimate emissions, by taking into account the change in carbon content from post-fire residues.

## Results

### Definitional preliminaries

Following the definitions from Surawski *et al*.^16^, we define ‘burnt' as fuel that has been thermally altered as a result of exposure to fire and either emitted to the atmosphere or left in the post-fire residue; and we define ‘consumed' as that component of the fuel that is emitted to the atmosphere as a result of exposure to fire. We follow the definition of Keane *et al*.^19^ that ‘fuels are the dead and live biomass available for fire ignition and combustion'.

### The basis of biased emission estimates

**Figure 1:**
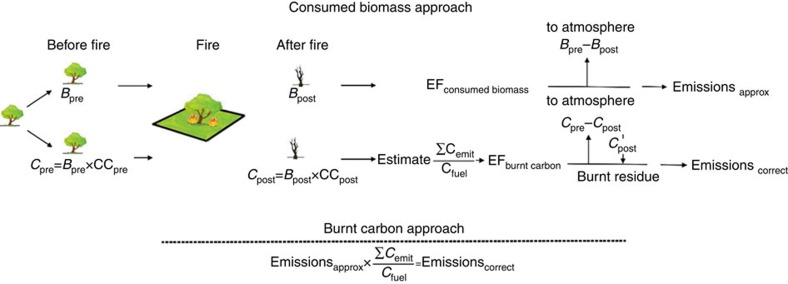
A diagrammatic illustration of how biased emission estimates from fire occur. A schematic of the two different ways the carbon mass balance technique has been interpreted and applied in vegetation fire emission studies. *C*_pre_ and *B*_pre_ denote pre-fire carbon and biomass fuel, CC_pre_ and CC_post_ represent pre- and post-fire carbon content, and *C*_post_ and *B*_post_ denote post-fire carbon and biomass fuel, respectively. Σ*C*_emit_/*C*_fuel_ represents the correction factor required for the burnt carbon accounting framework. Emissions_approx_ represents approximate emissions estimated using the consumed biomass approach and Emissions_correct_ represents emission estimates using the burnt carbon approach. EF denotes emissions factor with the estimate depending on what accounting framework is used (that is, consumed biomass or burnt carbon).

Since the publication of the CMB technique^15^, the method has been interpreted and applied in two different ways in the vegetation fire emission literature^17^,^20^. The first is the ‘consumed biomass' approach (Fig. 1, top pathway), which is based on the simplifying assumption that all burnt carbon is volatilized and emitted. We demonstrate below that this assumption is incorrect and leads to biased results. The second method, the ‘burnt carbon' approach, additionally takes into account changes between the pre- and post-fire carbon content of the fuel and is unbiased (Fig. 1, bottom pathway).

Both approaches have the same starting point, which is the amount of biomass fuel before burning (*B*_pre_, t ha^−1^ on a dry matter basis) and its carbon content (CC_pre_, %). In the absence of measurements of fuel carbon content, a carbon content of 50% is commonly assumed^18^, although a value of 45% has also been suggested^21^. The pre-burning carbon load (t ha^−1^ on a dry matter basis) can then be calculated from estimates of both *B*_pre_ and CC_pre_ (*C*_pre_=*B*_pre_ × CC_pre_). Both interpretations of the CMB technique rely on estimates of biomass fuel after burning (*B*_post_) to enable calculation of a combustion factor (CF=(*B*_pre_−*B*_post_)/*B*_pre_) (ref. 13). It is the subsequent analysis where the two interpretations of the CMB technique diverge and bias is introduced.

The ‘consumed biomass' approach is more commonly applied^21^,^22^,^23^,^24^,^25^ (and references therein) and does not require any further measurements after *B*_post_ has been calculated. In this case, estimation of an EF is based on consumed biomass only and reports EFs as the mass of a chemical species emitted per kilogram of dry fuel consumed. The ‘burnt carbon' approach is far less commonly applied^16^,^17^,^26^,^27^ and requires an estimate of the post-burning carbon content for burnt fuel that is not emitted to the atmosphere. Estimating CC_post_ enables the post-fire carbon load (*C*_post_=*B*_post_ × CC_post_) to be calculated. This in turn enables burnt carbon to be partitioned between that emitted to the atmosphere (Σ*C*_emit_=*C*_pre_−*C*_post_) and that remaining as post-burn combustion residue (*C*_post_, for example, charred material enriched in carbon; Fig. 1). Consequently, the second interpretation of the CMB technique is based on ‘burnt carbon' involving either atmospheric emission or resulting in an ashed or charred residue after exposure to fire (*C*_post_ in Fig. 1) with both of these ultimate fates being represented as a percentage of burnt carbon.

The explicit and incorrect assumption of the ‘consumed biomass' CMB method (top pathway in Fig. 1) is that all burnt carbon is volatilized and emitted to the atmosphere^20^,^25^,^28^. In contrast, the ‘burnt carbon' CMB method (bottom pathway in Fig. 1) correctly accounts for burnt carbon remaining as a residue after a vegetation fire. Many vegetation fire research studies have shown that not all burnt fuel carbon is volatilized and emitted to the atmosphere^17^,^26^,^29^,^30^,^31^,^32^,^33^,^34^. The need to take into account the full-carbon budget of vegetation fires has been raised previously^21^,^35^,^36^, yet vegetation fire emission studies generally do not measure CC_post_ (with CC_post_>CC_pre_ for charred post-fire material^30^,^31^,^34^), which precludes use of the ‘burnt carbon' CMB method and this leads to an overestimation bias of emissions for the ‘consumed biomass' method relative to the ‘burnt carbon' method.

To avoid this bias with the ‘consumed biomass' CMB approach, it is necessary to multiply its EF by the fraction of burnt fuel carbon emitted to the atmosphere, Σ*C*_emit_/*C*_fuel_ (ref. 16), where Σ*C*_emit_ is the mass of emitted carbon and *C*_fuel_ is the mass of fuel carbon burnt (Supplementary Table 1). Only if all burnt fuel carbon is emitted to the atmosphere (that is, Σ*C*_emit_/*C*_fuel_=1, which is very unlikely to be achieved in a real fire), would these two methods agree without inclusion of the correction factor. Otherwise, estimates of emissions using the ‘consumed biomass' approach will be overestimated (that is, relative to the ‘burnt' carbon approach), represented by the difference between complete volatilization and emission of carbon to the atmosphere, and the true fraction of carbon volatilized and emitted to the atmosphere (*ɛ*=1−Σ*C*_emit_/*C*_fuel_).

### Quantification of the correction factor

**Figure 2:**
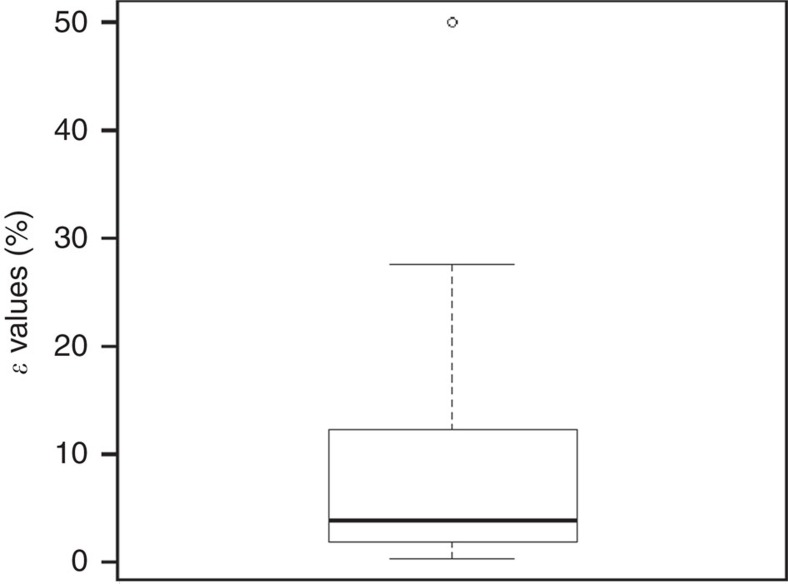
A box plot of the global *ɛ* value distribution based on our literature review. The *ɛ* value is the percentage of carbon exposed to fire (that is, burnt) that remains as a post-fire residue, based on 40 records^17^,^29^,^30^,^31^,^32^,^33^,^34^ (Supplementary Table 1). The five-number summary for *ɛ* is: minimum=0.4%, 25th percentile=1.98%, median=3.95%, 75th percentile=12.0%, maximum=50% (the single outlier is shown as an open circle). The interquartile range (IQR)=10.0.

To quantify the magnitude of this overestimation, we obtained empirical estimates from the literature^17^,^29^,^30^,^31^,^32^,^33^,^34^ (Supplementary Table 1) of the fraction of burnt fuel carbon emitted to the atmosphere or the fraction of burnt carbon retained in post-burn residues (and in one case both emissions and residues), to determine the likely values of *ɛ* in various vegetation types around the globe. *ɛ* values range from a minimum of 0.4% in a savanna ecosystem^34^ to a maximum of 50% in a temperate forest^34^. On the basis of 40 records (Fig. 2), the median *ɛ* value is 4.0% with the interquartile range (25th–75th percentiles) spanning the interval 2.0–12.0%.

## Discussion

In terms of estimating global emissions from vegetation fires, the widely used Global Fire Emissions Database^22^, and others such as the Fire INventory from NCAR^23^, and IPCC good practice guidelines for national greenhouse gas inventories^13^ rely on the ‘consumed biomass' approach for reporting EFs. If we use the median *ɛ* value of 4.0% and apply this to the globally averaged annual carbon emissions from 2001 to 2010 of 2.5 Pg (ref. 7), the overestimation of emissions (that is, for the ‘consumed biomass' approach compared with the ‘burnt carbon' approach) amounts to ∼100 Tg (1 Tg=1 × 10^12^ g) of carbon per year, with an uncertainty range (25th–75th percentiles) of 50–300 Tg. Furthermore, the ‘consumed biomass' approach (which assumes 100% volatilization of burnt carbon) precludes the ability to quantify the efficacy of any direct management of fire for post-fire residues for mitigating net vegetation fire emissions, such as the preferential firing of vegetation fuels for enhanced charred post-fire residues to increase sequestration in the pedosphere.

Altogether, the potential for a 50–300-Tg error in annual global vegetation fire emission estimates highlights that vegetation fire research should be conducted in a more coordinated manner^1^,^37^, to ensure the complete biogeochemistry of fire events are adequately represented. Despite recommendations in the literature^21^, measurements of CC_post_ are not routinely made in vegetation fire emission studies. This prevents Σ*C*_emit_/*C*_fuel_ from being calculated, leading to an overestimation of emissions for the ‘consumed biomass' approach relative to the ‘burnt carbon' approach. Furthermore, this overestimation means that the amount of carbon in burnt residues needs to be adjusted to maintain global carbon mass balance from vegetation fires. Apart from improving global vegetation fire emission estimates, it is envisaged that global studies of CC_post_ will broaden our understanding of ecosystem carbon fluxes due to fire, including the potential for managing burnt carbon residues to enhance ecosystem carbon storage and to spawn research efforts exploring the post-fire fate of carbon^34^,^35^,^36^,^38^. In particular, such efforts should estimate the global *ɛ* value with different fire behaviours and ecosystem types, as only a coarse estimate was obtained using current estimates in the literature. In conducting such research, however, it is important to realize that greater uncertainties are present for burnt areas, fuel load and combustion factors from global vegetation fires^39^.

## Methods

### EF reporting methods

Examples of studies that have adopted the approximate ‘consumed biomass' approach to emissions accounting from vegetation fires are ubiquitous^21^,^22^,^23^,^24^,^25^ (and references therein).

Examples of studies exploring the more complete ‘burnt carbon' approach are far less common^16^,^17^,^26^,^27^.

### EF reporting fundamentals

Estimating emissions from vegetation fires involves multiplying the amount of fuel consumed^6^ by an EF (ref. 24). The amount of fuel consumed is obtained by multiplying the area burnt (*A*) by the fuel load (*B*) and a combustion factor (CF). The basis of the CMB technique is that for the dominant carbonaceous species emitted in a plume (usually CO_2_, CO and CH_4_), excess mixing ratios (that is, molar concentrations above background) are measured. Then, emission ratios are calculated by normalizing (that is, dividing) the excess mixing ratio of the species of interest to the excess mixing ratio of a reference gas, or in some cases the sum of the excess mixing ratios of total emitted carbon^40^. An EF is then obtained by dividing the emission ratio for the species of interest by that of the emitted and detected carbon

### Conversion of the consumed biomass method to burnt carbon

Using the ‘consumed biomass' interpretation of an EF (an approximation that assumes that all fuel carbon is volatilized and emitted to the atmosphere), emissions for the *X*th carbon-based species (*E*_*X*_) can be calculated by multiplying the area burnt, the fuel load and the combustion and EFs together via:


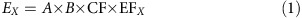


In equation (1), EF_*X*_, reported on a kg species *X* per kg consumed basis, is defined by^18^:


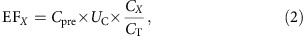


where *U*_C_ is a conversion factor used to convert between atomic and molar masses, *C*_*X*_ is the number of moles of species *X* and *C*_T_ is the total number of carbon moles emitted to the atmosphere.

Assuming that CO_2_ is used as a reference gas for the normalization of emission ratios (that is, other choices include CO or CH_4_ (ref. 16) or total emitted carbon^40^), *C*_*X*_*/C*_T_ is given by:


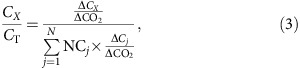


where Δ*C*_*X*_ represents the difference between the plume and ambient mixing ratios for species *X*, and NC_*j*_ represents the number of carbon atoms in species *j*, with summation being performed across the total number of carbon species *N*.

Alternatively, using the ‘burnt carbon' interpretation, emissions for the *X*th (that is, carbon based) species are given by:


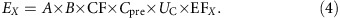


In equation (4), assuming again that CO_2_ is used as a reference gas, EF_*X*_ (defined as the percentage of burnt carbon emitted as species *X*) is defined by^17^:


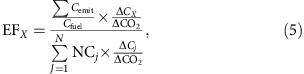


where Σ*C*_emit_ is the mass of fuel carbon emitted to the atmosphere from *j* different carbon species, and *C*_fuel_ is the mass of fuel carbon burnt.

Note that:


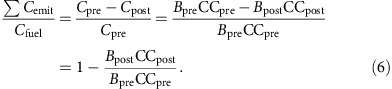


Consequently, the only way that the equation (1) can equal equation (4) is if equation (2) is multiplied by Σ*C*_emit_/*C*_fuel_ (ref. 16). The presentation here focused on carbon-based species, but similar calculations can be performed for nitrogen-based species (such as N_2_O).

### Global estimates of the correction factor

A broad literature search was conducted using the Web of Science, Scopus and Google Scholar databases to parameterize this quantity. Literature searches were motivated by listings of major field-based vegetation fire emission campaigns commencing in the 1980s and ending with the Fire Lab at Missoula Experiments (Supplementary Table 1). On the basis of our literature search, in Supplementary Table 1, we parameterise the global *ɛ* value for different ecosystems and fuel types, as well as for different fire types, fuel sampling methods, carbon residue sampling methods and carbon residue quantification methods. A statistical summary of our *ɛ* value database is provided in Fig. 2 and its caption.

## Additional information

**How to cite this article:** Surawski, N. C. *et al*. Incorrect interpretation of carbon mass balance biases global vegetation fire emission estimates. *Nat. Commun.* 7:11536 doi: 10.1038/ncomms11536 (2016).

## Supplementary Material

Supplementary Information Supplementary Table 1 and Supplementary References
