# Author Correction: Incorrect interpretation of carbon mass balance biases global vegetation fire emission estimates

**DOI:** 10.1038/s41467-023-40256-3

**Published:** 2023-08-07

**Authors:** N. C. Surawski, A. L. Sullivan, S. H. Roxburgh, C. P. Mick Meyer, P. J. Polglase

**Affiliations:** 1 CSIRO Agriculture, GPO Box 1700, Canberra, Acton, 2601 ACT Australia; 2 CSIRO Land and Water, GPO Box 1700, Canberra, Acton, 2601 ACT Australia; 3 CSIRO Oceans and Atmosphere, 107–121 Station Street, Aspendale, VIC 3195 Australia; 4 Present Address: Energy, Environment and Water Research Center (EEWRC), The Cyprus Institute, PO Box 27456, Nicosia, 2121 Cyprus; 5 Present Address: Fenner School of Environment & Society, Australian National University, Linnaeus Way, Acton, ACT 2601 Australia

Correction to: *Nature Communications* 10.1038/ncomms11536, published online 05 May 2016

The original version of this Article contained an error in Equation 2. Equation 2 incorrectly read: ${{{{\rm{EF}}}}}_{{{{\rm{X}}}}}={{{{\rm{C}}}}}_{{{{\rm{pre}}}}}\times {{{{\rm{U}}}}}_{{{{\rm{C}}}}}\times \frac{{C}_{X}}{{C}_{T}}$

The correct form of Equation 2 is: ${{{{\rm{EF}}}}}_{{{X}}}={{{CC}}}_{{{{\rm{F}}}},{{{\rm{pre}}}}}\times {{{U}}}_{{{{\rm{C}}}}}\times \frac{{C}_{X}}{{C}_{{{\rm{T}}}}}$

The original version of this Article contained an error in Equation 4. Equation 4 incorrectly read: E_X_ = A × B × CF × C_pre_ × U_C_ × EF_X_

The correct form of Equation 4 is: *E*_*X*_ = *A* × *B* × CF × *CC*_F,pre_ × *U*_C_ × EF_*X*_

The original version of this Article contained an error in the description of equation 2 in the Methods section, which incorrectly read ‘where *U*_C_ is a conversion factor used to convert between atomic and molar masses, *C*_*X*_ is the number of moles of species *X* and *C*_T_ is the total number of carbon moles emitted to the atmosphere.’

The correct version replaces this with ‘where *CC*_F,pre_ is the pre-fire carbon fraction of the fuel, *U*_C_ is a conversion factor used to convert between atomic and molar masses, *C*_*X*_ is the number of moles of species *X* and *C*_T_ is the total number of carbon moles emitted to the atmosphere.’.

This has been corrected in the PDF and HTML versions of the Article.

