# Supplementary material for: Incorrect interpretation of carbon mass balance biases global vegetation fire emission estimates
Source: Nat Commun. 2016 May 5;7:11536. doi: 10.1038/ncomms11536 (PMC4858743; doi:10.1038/ncomms11536)
Supplement: Supplementary Information — Supplementary Table 1 and Supplementary References [file ncomms11536-s1.pdf]

Supplementary Table 1.

Study	Ecosystem/fuel type	Fire type	Pre/post fire fuels sampled	Carbon (C) residue sampling methods	C residue quantification method	ε value (%)
Alexis et al. (2007)[1]	Sub-tropical	Prescribed fire	Leaves, stems and litter	Gravimetric	Elemental analyser	3.4
Barbosa and Fearnside (1996)[2]	Amazonian pasture	Prescribed slash and burn fire	Total above ground biomass	Volumetric (for coarse woody debris) and gravimetric (not coarse woody debris) sampling	Combustion at 1100 ^{o}C followed by electrical conductivity measurement	4.1
Brewer et al. (2013)[3]	Temperate	Laboratory fuel beds	Surface fuels < 7.6 cm diameter	Gravimetric	High temperature combustion	8.1
Carvahlo et al. (2011)[4]	Sub-tropical	Prescribed fires	Wood blocks	Visual selection of charred wood pieces	Gravimetric charcoal content determination	3.1
Clay and Worrall (2011)[5]	Temperate peatland	Wildfire	Total above ground biomass excluding standing wood	Gravimetric	C combustion system	14
Czimczik et al. (2003)[6]	Boreal forest	Wildfire	Whole forest floor	Gravimetric	Benzenepolycarboxylic acids as molecular markers	0.7
Eckmeier et al. (2007)[7]	Temperate	Experimental slash and burn	Slashed fuels < 10 cm in diameter	Gravimetric	Dry combustion in an elemental analyser	8.1ƚ
Fearnside et al. (1993)[8]	Tropical rainforest	Prescribed slash and burn fire	Total above ground biomass	Visual and gravimetric	Assumed charcoal carbon content	2.7
Fearnside et al. (1999)[9]	Tropical rainforest	Prescribed slash and burn fire	Total above ground biomass	Gravimetric	Assumed charcoal carbon content	1.3
Fearnside et al. (2001)[10]	Tropical rainforest	Prescribed slash and burn fire	Total above ground biomass	Gravimetric	Assumed charcoal carbon content	1.8

Fearnside et al. (2007)[11]	Tropical	Prescribed slash and burn fire	Total above ground biomass	Gravimetric	Assumed C value	1.9
Finkral et al. (2012)[12]	Temperate	Prescribed slash pile burn	Logged trees < 12.7 cm in diameter piled with other logging residues	Volumetric - converted to gravimetric using assumed wood density	Elemental analyser	3.1
Forbes et al. (2006)[13]	Sub-tropical grassland	Savanna fire	Not mentioned	Not mentioned	Carbon-to-Silicon ratios using ^{13}C-NMR spectroscopy	2.5
Graça et al. (1999)[14]	Tropical rainforest	Experimental slash and burn fire	Total above ground biomass	Gravimetric and volumetric (for wood)	Carbon analyser	3.2
Hurst et al. (1994)[15]	Tropical Savanna	Prescribed savanna fires	Understorey and tree litter, grass, leaves and twigs	Gravimetric	Total combustion process	3.7*
Kauffman et al. (1995)[16]	Tropical rainforest	Prescribed slash and burn fire	Total above ground biomass	Ash depth measurement	Ash depth converted to bulk density using known volume and mass and C quantification performed using Induction furnace elemental analyser	1.3
Kauffman et al. (1998)[17]	Amazonian pasture	Prescribed slash and burn fire	Total above ground biomass	Gravimetric	Induction furnace elemental analyser	1.9
Kulbusch and Crutzen 1995[18]	Deciduous wood	Laboratory backing fire	Sampling for burn table residues	Gravimetric	Solvent extraction, thermal treatment at 340 ^{o}C and three stage elemental analysis	5.4

Kulbusch and Crutzen 1995	Hay	Laboratory backing fire	Sampling for burn table residues	Gravimetric	Solvent extraction, thermal treatment at 340 ºC and three stage elemental analysis	4.6
Kulbusch and Crutzen 1995	Needle litter	Laboratory backing fire	Sampling for burn table residues	Gravimetric	Solvent extraction, thermal treatment at 340 ºC and three stage elemental analysis	6.2
Kulbusch and Crutzen 1995	Pine needle	Laboratory backing fire	Sampling for burn table residues	Gravimetric	Solvent extraction, thermal treatment at 340 ºC and three stage elemental analysis	2.9
Kulbusch and Crutzen 1995	Savanna grass African	Laboratory backing fire	Sampling for burn table residues	Gravimetric	Solvent extraction, thermal treatment at 340 ºC and three stage elemental analysis	1.7
Kulbusch and Crutzen 1995	Savanna grass Venezuela	Laboratory backing fire	Sampling for burn table residues	Gravimetric	Solvent extraction, thermal treatment at 340 ºC and three stage elemental analysis	3.8
Kulbusch and Crutzen 1995	Straw	Laboratory backing fire	Sampling for burn table residues	Gravimetric	Solvent extraction, thermal treatment at 340 ºC and three stage elemental analysis	1.9
Kulbusch and Crutzen 1995	Needle litter	Laboratory heading fire	Sampling for burn table residues	Gravimetric	Solvent extraction, thermal treatment at 340 ºC and three stage elemental analysis	17.2
Kulbusch and Crutzen 1995	Needle litter Phillipines	Laboratory heading fire	Sampling for burn table residues	Gravimetric	Solvent extraction, thermal treatment at 340 ºC and three stage elemental analysis	17.5

Kulbusch and Crutzen 1995	Savanna grass Venezuela	Laboratory heading fire	Sampling for burn table residues	Gravimetric	Solvent extraction, thermal treatment at 340 ^{o}C and three stage elemental analysis	4.5
Kulbusch and Crutzen 1995	Straw	Laboratory heading fire	Sampling for burn table residues	Gravimetric	Solvent extraction, thermal treatment at 340 ^{o}C and three stage elemental analysis	1.2
Kulbusch et al. 1996[19]	Savanna	Experimental savanna fire (heading fire)	Grass, litter and herbs	Gravimetric	Solvent extraction, thermal treatment at 340 ^{o}C and three stage elemental analysis	9.2
Kulbusch et al. 1996	Savanna	Experimental savanna fire (backing fire)	Grass, litter and herbs	Gravimetric	Solvent extraction, thermal treatment at 340 ^{o}C and three stage elemental analysis	13.1
Righi et al. (2009)[20]	Tropical	Prescribed slash and burn fire	Total above ground biomass	Volumetric - converted to gravimetric using assumed charcoal density	Carbon analyser using dry combustion method	6
Rumpel et al. (2009)[21]	Savanna	Prescribed slash and burn fire	Straw	Gravimetric	Carbon analyser using dry combustion method	0.4
Russell-Smith et al. (2009)[22]	Tropical Savanna	Prescribed savanna fires	Fine, coarse and heavy fuels plus shrubs	Gravimetric	Assumed C value	11.6
Saiz et al. (2015)[23]	Savanna grassland	Experimental fires in the field	Grass, litter and shrubs	Gravimetric	Hydrogen pyrolysis followed by Elemental Analysis Isotope Ratio Mass Spectrometry	15.8

Saiz et al. (2015)	Shrub-rich savanna woodland	Experimental fires in the field	Grass, litter and shrubs	Gravimetric	Hydrogen pyrolysis followed by Elemental Analysis Isotope Ratio Mass Spectrometry	16.3
Saiz et al. (2015)	Savanna woodland	Experimental fires in the field	Grass, litter and shrubs	Gravimetric	Hydrogen pyrolysis followed by Elemental Analysis Isotope Ratio Mass Spectrometry	13.8
Saiz et al. (2015)	Tall savanna woodland	Experimental fires in the field	Grass, litter and shrubs	Gravimetric	Hydrogen pyrolysis followed by Elemental Analysis Isotope Ratio Mass Spectrometry	17.3
Santín et al. (2015)[24]	Boreal forest	Experimental fire in the field	Forest floor fuels, downed wood and overstorey bark and needles	Fuel type dependent methods	High temperature combustion and conversion to CO_2 plus testing for carbonates using HCl	27.6
Tinker and Knight (2000)[25]	Temperate forest	Crown fire	Coarse woody debris > 7.5 cm in diameter	Visual	Volumetric to gravimetric conversion using wood density values	50ł
Worrall et al. (2013)[26]	Temperate	Prescribed	Total above ground biomass excluding standing wood	Gravimetric	C combustion system	2

Supplementary Table 1. Sources of data used to parameterise ε values globally.

* Speciated carbon emissions measured in addition to carbon residue.
ł No multi-plot/site averaging of carbon residues was performed.

Supplementary References

1. Alexis, M. A. *et al*. Fire impact on C and N losses and charcoal production in a scrub oak ecosystem. *Biogeochemistry* **82**, 201-216, doi:10.1007/s10533-006-9063-1 (2007).
2. Barbosa, R. I. & Fearnside, P. M. Pasture burning in Amazonia: Dynamics of residual biomass and the storage and release of aboveground carbon. *Journal of Geophysical Research-Atmospheres* **101**, 25847-25857, doi:10.1029/96JD02090 (1996).
3. Brewer, N. W *et al*. Fuel moisture influences on fire-altered carbon in masticated fuels: An experimental study. *Journal of Geophysical Research-Biogeosciences* **118**, 30-40, doi:10.1029/2012JG002079 (2013).
4. Carvalho, E. O., Kobziar, L. N. & Putz, F. E. Fire ignition patterns affect production of charcoal in southern forests. *International Journal of Wildland Fire* **20**,474-477, doi:10.1071/WF10061 (2011).
5. Clay, G. D. & Worrall, F. Charcoal production in a UK moorland wildfire - How important is it? *Journal of Environmental Management* **92**, 676-682, doi:10.1016/j.jenvman.2010.10.006 (2011).
6. Czimczik, C. I., Preston, C. M., Schmidt, M. W. I. & Schulze, E. – D. How surface fire in Siberian Scots pine forests affects soil organic carbon in the forest floor: Stocks, molecular structure, and conversion to black carbon (charcoal). *Global Biogeochemical Cycles* **17**, doi:10.1029/2002GB001956, (2003).
7. Eckmeier, E. *et al*. Conversion of biomass to charcoal and the carbon mass balance from a slash-and-burn experiment in a temperate deciduous forest. *The Holocene* **17**, 539-542, doi:10.1177/0959683607077041 (2007).
8. Fearnside, P. M., Leal, N. Jr & Fernandes, F. M. Rainforest Burning and the Global Carbon Budget: Biomass, Combustion Efficiency, and Charcoal Formation in the Brazilian Amazon. *Journal of Geophysical Research-Atmospheres* **98**, 16733-16743, doi:10.1029/93JD01140 (1993).
9. Fearnside, P. M., Graça, P. M. L. D., Leal, N., Rodrigues, F. J. A. & Robinson, J. M. Tropical forest burning in Brazilian Amazonia: measurement of biomass loading, burning efficiency and charcoal formation at Altamira, Pará. *Forest Ecology and Management* **123**, 65-79, doi:10.1016/S0378-1127(99)00016-X (1999).
10. Fearnside, P. M., Graça, P. M. L. D., & Rodrigues, F. J. A. Burning of Amazonian rainforests: burning efficiency and charcoal formation in forest cleared for cattle pasture near Manaus, Brazil. *Forest Ecology and Management* **146**, 115-128, doi:10.1016/S0378-1127(00)00450-3 (2001).
11. Fearnside, P. M., Barbosa, R. I. & Graça, P. M. L. D. Burning of secondary forest in Amazonia: Biomass, burning efficiency and charcoal formation during land preparation for agriculture in Apiaú, Roraima, Brazil. *Forest Ecology and Management* **242**, 678-687, doi:10.1016/j.foreco.2007.02.002 (2007).
12. Finkral, A. J., Evans, A. M., Sorensen, C. D. & Affleck, D. L. R. Estimating consumption and remaining carbon in burned slash piles. *Canadian Journal of Forest Research* **42**, 1744-1749, doi:10.1139/X2012-112 (2012).
13. Forbes, M. S., Raison, R. J. & Skjemstad, J. O. Formation, transformation and transport of black carbon (charcoal) in terrestrial and aquatic ecosystems. *Science of the Total Environment* **370**, 190-206, doi:10.1016/j.scitotenv.2006.06.007 (2006).
14. Graça, P. M. L. D. A., Fearnside, P. M. & Cerri, C. C. Burning of Amazonian forest in Ariquemes, Rondônia, Brazil: biomass, charcoal formation and burning efficiency. *Forest Ecology and Management* **120**, 179-191, doi:10.1016/S0378-1127(98)00547-7 (1999).
15. Hurst, D. F., Griffith, D. W. T. & Cook, G. D. Trace gas emissions from biomass burning in tropical Australian savannas. *Journal of Geophysical Research-Atmospheres* **99**, 16441-16456, doi:10.1029/94jd00670 (1994).
16. Kauffmann, J. B., Cummings, D. L., Ward, D. E. & Babbitt, R. Fire in the Brazilian Amazon: 1. Biomass, nutrient pools, and losses in slashed primary forests. *Oecologia* **104**, 397-408, doi:10.1007/BF00341336 (1995).
17. Kauffmann, J. B., Cummings, D. L., & Ward, D. E. Fire in the Brazilian Amazon 2. Biomass, nutrient pools and losses in cattle pastures. *Oecologia* **113**, 415-427, doi:10.1007/s004420050394 (1998).
18. Kuhlbusch, T. A. J., & Crutzen, P. J. Toward a global estimate of black carbon in residues of vegetation fires representing a sink of atmospheric CO_2 and a source of O_2. *Global Biogeochemical Cycles* **9**, 491-501, doi:10.1029/95GB02742 (1995).
19. Kuhlbusch, T. A. J. *et al*. Black carbon formation by savanna fires: Measurements and implications for the global carbon cycle. *Journal of Geophysical Research-Atmospheres* **101**, 23651-23655, doi:10.1029/95JD02199 (1996).

20. Righi, C. A., Graça, P. M. L. D., Cerri, C. C., Feigl, B. J. & Fearnside, P. M. Biomass burning in Brazil's Amazonian ''arc of deforestation'': Burning efficiency and charcoal formation in a fire after mechanized clearing at Feliz Natal, Mato Grosso. *Forest Ecology and Management* **258**, 2535-2546, doi:10.1016/j.foreco.2009.09.010 (2009).
21. Rumpel, C., Ba, A., Darboux, F., Chaplot, V. & Planchon, O. Erosion budget and process selectivity of black carbon at meter scale. *Geoderma* **154**, 131-137, doi:10.1016/j.geoderma.2009.10.006 (2009).
22. Russell-Smith, J. *et al.* Improving estimates of savanna burning emissions for greenhouse accounting in northern Australia: limitations, challenges, applications. *International Journal of Wildland Fire* **18**, 1-18, doi:10.1071/wf08009 (2009).
23. Saiz, G. *et al.* Pyrogenic carbon from tropical savanna burning: production and stable isotope composition. *Biogeosciences* **12**, 1849-1863, doi:10.5194/bg-12-1849-2015 (2015).
24. Santín, C., Doerr, S. H., Preston, C. M. & Gonzalez-Rodriguez, G. Pyrogenic organic matter production from wildfires: a missing sink in the global carbon cycle. *Global Change Biology* **21**, 1621-1633, doi:10.1111/gcb.12800 (2015).
25. Tinker, D. B. & Knight, D. H. Coarse woody debris following fire and logging in Wyoming lodgepole pine forests. Ecosystems **3**, 472-483, doi:10.1007/s100210000041 (2000).
26. Worrall, F. Clay, G. D. & May, R. Controls upon biomass losses and char production from prescribed burning on UK moorland. *Journal of Environmental Management* **120**, 27-36, doi:10.1016/j.jenvman.2013.01.03027-36 (2013).
